# Synergistic Protection of Vitamin B Complex and Alpha-Lipoic Acid Against Hepatic Ischemia–Reperfusion Injury: Boosting Antioxidant Defenses in Rats

**DOI:** 10.3390/cimb46120810

**Published:** 2024-11-28

**Authors:** Fatih Seğmen, Semih Aydemir, Onur Küçük, Ümit Murat Parpucu, Recep Dokuyucu

**Affiliations:** 1 Department of Intensive Care Unit, Ankara City Hospital, 06800 Ankara, Türkiye; fatih.segmen1@saglik.gov.tr; 2 Department of Anesthesiology and Reanimation, Yenimahalle Training and Research Hospital, University of Yıldırım Beyazit, 06760 Ankara, Türkiye; semih.aydemir@saglik.gov.tr; 3 Department of Anesthesiology and Reanimation, Ankara Atatürk Sanatorium Training and Research Hospital, University of Health Sciences, 90203 Ankara, Türkiye; onur.kucuk4@saglik.gov.tr; 4 Department of Anesthesiology and Reanimation, Gülhane Faculty of Health Sciences, University of Health Sciences, 06010 Ankara, Türkiye; umitmurat.parbucu@sbu.edu.tr; 5 Department of Physiology, Medical Specialization Training Center (TUSMER), 06230 Ankara, Türkiye

**Keywords:** liver, ischemia–reperfusion injury, alpha-lipoic acid, vitamin B complex, oxidative stress

## Abstract

This study aimed to investigate the protective effects of vitamin B complex and alpha-lipoic acid (ALA) pre-treatments on hepatic ischemia–reperfusion injury (IRI) in rats, focusing on their potential to enhance antioxidant defense mechanisms and reduce post-ischemic liver damage. Thirty male Wistar albino rats were divided into four groups: sham group (n = 10), IRI group (n = 10), vitamin B group (n = 10), vitamin B + ALA group (n = 10). In the IRI, vitamin B, and vitamin B + ALA groups, the rats underwent 45 min of hepatic ischemia followed by 60 min of reperfusion. Serum levels of aspartate aminotransferase (AST), alanine aminotransferase (ALT), urea, creatinine, and lactate dehydrogenase (LDH) were measured. Additionally, serum total antioxidant status (TAS) and total oxidant status (TOS) were assessed, and the oxidative stress index (OSI) was calculated. Liver tissue samples were collected for morphological evaluation. In the vitamin B and vitamin B + ALA groups, ALT, AST, urea, creatinine and LDH levels were better compared with the IRI group but the difference was statistically significant for only LDH levels in the vitamin B group and ALT, urea, and LDH levels in the vitamin B + ALA group (*p* < 0.05). The lowest TOS and OSI levels were reported in the vitamin B and vitamin B + ALA groups and these groups had statistically significantly higher TAS compared with the sham and IRI groups (*p* < 0.05). Our findings suggest that a vitamin B complex alone or a vitamin B complex + ALA combination reduces post-ischemic hepatic injury by enhancing the anti-oxidative status. The low dose of ALA may be a co-factor in these results and studies with larger doses of ALA are required to determine its effects on IRI of the liver.

## 1. Introduction

Ischemia–reperfusion injury (IRI) of the liver, caused by a temporary cessation of blood flow followed by its restoration, primarily affects oxygen-dependent cells and is associated with significant clinical challenges, including elevated morbidity and mortality rates [1]. Clinically, liver IRI manifests through increased liver enzymes, biliary strictures, liver failure, and potentially multi-organ dysfunctions, complicating patient outcomes following surgeries such as liver transplantation or resection [2].

Central to the pathogenesis of IRI are reactive oxygen species (ROS) and oxidative stress. ROS, which include highly reactive molecules such as superoxide and hydroxyl radicals, are key contributors to cellular damage during IRI. These radicals exacerbate microvascular permeability, leading to interstitial edema and inflammation. Kupffer cells, the resident macrophages of the liver, are activated in response to ROS and further propagate oxidative stress and inflammation by releasing additional ROS and pro-inflammatory cytokines. This creates a vicious cycle of cellular damage and inflammation, ultimately facilitating pathways leading to cell death, including apoptosis and necrosis [1].

Reactive oxygen species (ROS) and oxidative stress play a crucial role in initiation and continuation of IRI of the liver. As in any organ or tissue, in the liver, ROS accumulation has been reported to facilitate the cell death pathways [3]. Given the central role of oxidative stress in IRI, various strategies have been explored to mitigate its effects. In the prevention of IRI in the liver, especially during surgeries or transplantation; ischemic preconditioning (IP) and some different antioxidant agents have been suggested by way of reducing oxidative stress [4]. Ischemic preconditioning (IP), a technique involving brief episodes of ischemia and reperfusion before the main ischemic event, has been shown to reduce oxidative damage and improve tissue resilience. Additionally, the administration of antioxidant agents has been proposed as a therapeutic strategy to counteract ROS and their deleterious effects [5,6,7].

Alpha-lipoic acid (ALA) is a potent antioxidant that is unique due to its solubility in both lipid and aqueous environments, making it particularly effective in various cellular compartments [8,9,10]. ALA is essential for mitochondrial bioenergetic processes and has been demonstrated to directly scavenge ROS, regenerate other antioxidants such as vitamin C and glutathione, and maintain cellular redox balance. The therapeutic potential of ALA has been extensively studied in conditions characterized by high oxidative stress, including diabetes, atherosclerosis, neurodegenerative diseases, and IRI [10,11,12].

Furthermore, different forms of vitamin B, including vitamin B12 (cobalamin), riboflavin, and thiamine, are known for their anti-inflammatory and antioxidant properties [13,14,15]. These vitamins play crucial roles in cellular metabolism and energy production, which are vital under conditions of stress and injury. For instance, vitamin B12 is involved in the methionine synthase pathway, which is crucial for DNA synthesis and repair, and has been shown to reduce oxidative stress and inflammation. Riboflavin and thiamine also contribute to cellular energy production and have been reported to offer protective effects against oxidative damage in various tissues, including the liver [16,17]. Despite these individual findings, the combined effect of a vitamin B complex in preventing liver IRI has not been thoroughly investigated. Vitamin B complexes, which include a combination of several B vitamins, might provide synergistic benefits by targeting multiple pathways involved in oxidative stress and inflammation.

Ischemic preconditioning (IP) has been widely recognized as a protective strategy against ischemia–reperfusion injury (IRI) through its hormetic effects, which involve a mild stress stimulus that induces cellular resistance to subsequent, more severe stress. A key mechanism in IP is the activation of antioxidant response element (ARE)-driven pathways. These pathways are mediated by nuclear factor erythroid 2-related factor 2 (Nrf2), which translocates to the nucleus upon oxidative stress, upregulating the expression of endogenous antioxidants such as superoxide dismutase, catalase, and glutathione peroxidase. This process primes cells to mitigate oxidative damage and inflammation during reperfusion. The use of exogenous antioxidants, such as alpha-lipoic acid and vitamin B complex, aims to mimic these hormetic effects by directly neutralizing reactive oxygen species (ROS) and enhancing the endogenous antioxidant defense systems. Thus, understanding the mechanistic overlap between IP and antioxidant administration can provide deeper insights into strategies to reduce hepatic IRI [18,19,20,21].

In this experimental study, we aimed to elucidate the histopathological effects of vitamin B complex and ALA pretreatments in preventing hepatic IRI. Additionally, we assessed their impact on the oxidative status in a rat model. By exploring these potential therapeutic strategies, we hope to contribute to the development of more effective treatments for hepatic IRI, ultimately improving clinical outcomes for patients undergoing liver surgeries.

## 2. Materials and Methods

### 2.1. Study Design

Male Wistar albino rats weighing 300–400 g were used in this study. All animals had access to water and rat chow ad libitum (Global Diet Harlan). There were 4 experimental groups studied: Group 1 (sham group): only liver resection was performed (n = 10); Group 2 (IRI group): 45 min ischemia with 60 min of reperfusion after liver resection (n = 10); Group 3 (vitamin B group): vitamin B complex (Bemiks ampul) 0.05 mL/kg intraperitoneally was administered 15 min prior to ischemia and immediately before the reperfusion period (n = 10); Group 4 (Vitamin B + ALA group): vitamin B complex 0.05 mL/kg and alpha-lipoic acid (ALA) 100 mg/kg were administered intraperitoneally 15 min prior to ischemia and immediately before the reperfusion period (n = 10).

The vitamin B complex (Bemiks^®^, Zentiva, Istanbul, Turkey) formulation contained the following per 2 mL: 25 mg of thiamine hydrochloride (vitamin B1), 2.734 mg of riboflavin phosphate (vitamin B2), 5 mg of pyridoxine (vitamin B6), 15 mcg of vitamin B12, 50 mg of niacinamide, and 17.2 mg of D-panthenol.

The doses and timing of administration for the vitamin B complex and alpha-lipoic acid (ALA) in this study were determined based on previous experimental studies and pharmacokinetic considerations. The dose of vitamin B complex (0.05 mL/kg) aligns with prior research investigating its antioxidant and protective effects in ischemia–reperfusion models [13,22]. Similarly, the dose of ALA (100 mg/kg) was selected based on its established efficacy in mitigating oxidative stress and inflammation in animal models of IRI, as reported by Dulundu et al. and Ren et al. [23,24]. ALA’s pharmacokinetics in rats suggest rapid absorption and peak plasma concentrations within 15–30 min after intraperitoneal administration [8,25]. Administering both agents 15 min prior to ischemia and immediately before reperfusion ensures their presence during the critical phases of oxidative damage, thereby maximizing their therapeutic potential. These timings are consistent with studies indicating the importance of early antioxidant intervention to prevent the cascade of ROS-mediated injury during ischemia–reperfusion [9,21].

### 2.2. Experimental Procedures

Anesthesia was induced with intraperitoneal administration of xylazine (Rompun^®^, Bayer, Germany) at a dose of 10 mg/kg, followed by ketamine (Ketalar^®^, Pfizer, New York, NY, USA) at a dose of 10 mg/kg. Afterward, the abdominal cavity was accessed via a midline incision. Ischemia was induced by applying a microvascular clamp to the portal triad, causing hepatic ischemia for 45 min. This was followed by a 60-min reperfusion period, initiated by the removal of the clamp. The abdominal cavity was then closed using running sutures, and no additional fluid resuscitation was administered. Although the initial bolus of ketamine and xylazine was sufficient for the induction of anesthesia, the depth of sedation was regularly assessed using the absence of withdrawal reflexes (e.g., toe pinch test) and corneal reflex. If the animals showed signs of inadequate sedation during the surgical procedure, supplemental doses of anesthesia were administered intraperitoneally to maintain the proper depth of anesthesia. For analgesia, care was taken to minimize pain and discomfort by administering preoperative and postoperative analgesics, such as meloxicam (1 mg/kg subcutaneously), immediately after the surgical procedures. The animals were monitored closely for sedation levels during the entire experiment, and supplemental doses of anesthesia and analgesics were administered as needed to ensure that no animal experienced pain or distress. Finally, euthanasia was performed at the end of the experiment using a method consistent with AVMA guidelines for the euthanasia of animals. In this study, the animals were euthanized by intraperitoneal administration of a high dose of pentobarbital sodium (150 mg/kg), ensuring rapid and humane termination without distress. After 4 h of reperfusion, the animals were euthanized, and liver tissue and plasma samples were collected for subsequent analysis [13,26,27,28].

### 2.3. Measurements

Blood samples were centrifuged at 1500× *g* for 15 min and the serum samples were stored at −80 °C for biochemical analyses. Serum aspartate amino transferase (AST), alanine amino transferase (ALT) activities, urea and creatinine and LDH (lactate dehydrogenase) levels were measured by spectrophotometric methods in an auto-analyzer (Architect c8000, Clinical Chemistry Analyzer, Abbott, Abbott Park, IL, USA). Serum total antioxidant status (TAS) and total oxidant status (TOS) were quantified using commercially available kits from Rel Assay Diagnostic (Gaziantep, Turkey). The TAS assay measures the cumulative effect of all antioxidants present in the serum and is expressed as mmol Trolox equivalents per liter. The TOS assay, on the other hand, quantifies the total amount of oxidants, mainly hydrogen peroxide (H_2_O_2_) equivalents, present in the serum and is expressed as µmol H_2_O_2_ equivalents per liter. The oxidative stress index (OSI) was calculated to provide a comprehensive assessment of oxidative stress. The OSI is a ratio of the TOS to TAS, calculated using the following formula: OSI (arbitrary unit) = TOS (µmol H_2_O_2_ Eq/L)/TAS (mmol Trolox Eq/L) [9,29,30,31,32].

Formalin-fixed hepatic tissue samples were embedded in paraffin, and 5 μm sections were cut. Replicate sections were stained with hematoxylin and eosin (H&E) for the evaluation of morphologic features. Histopathological evaluations were conducted by a blinded pathologist using established criteria for ischemia–reperfusion injury. Cellular swelling, congestion, polymorph nuclear leukocyte infiltration, apoptosis, necrosis, and degeneration (including steatosis) were scored semi-quantitatively on a scale of 0 to 3: 0 = absent, 1 = mild, 2 = moderate, and 3 = severe, as described in previous studies evaluating hepatic ischemia–reperfusion injury [23,33]. This approach ensured standardized assessment and reproducibility of the findings.

### 2.4. Statistical Analysis

All statistical analyses were performed using SPSS software (Statistical Package for the Social Sciences version 21, Chicago, IL, USA). According to the power analysis (effect size: 2, α: 0.05, and power: 0.95), we calculated that the number of rats in the groups should be at least 7 (G*Power 3.1.19.2). To account for potential dropouts or unexpected losses during the experiment and to improve the reliability of the results, we decided to use 10 animals per group, aligning with recommendations in preclinical research to enhance statistical robustness [34]. For group comparisons, the statistical analysis has been revised to address the methodological concerns. Initially, data were analyzed using one-way analysis of variance (ANOVA) to detect overall differences among the four groups. For parameters with significant results in ANOVA (*p* < 0.05), post hoc (Tukey’s test) multiple comparison tests were applied to identify specific group differences. Due to the high variability observed in the sham group for some parameters, additional non-parametric analyses (Kruskal–Wallis test) were also performed to validate the results, followed by pairwise comparisons using the Mann–Whitney U test with Bonferroni correction. This multi-step approach ensures the statistical rigor of the study and accounts for variability that could obscure significant differences. All data are expressed as mean ± standard deviation (SD). A *p*-value of <0.05 was considered statistically significant.

## 3. Results

The biochemical parameters measured in this study demonstrated significant differences among the groups. The results are summarized in Table 1. AST levels were markedly elevated in the IRI group (1573.0 ± 1437.17) relative to the sham group (121.28 ± 44.35) (*p* < 0.01). The vitamin B group (1193.37 ± 493.40) and the vitamin B + ALA group (1002.62 ± 133.84) exhibited significantly lower AST levels compared to the IRI group (*p* < 0.05). The IRI group showed significantly increased urea levels (28.75 ± 2.98) compared to the sham group (18.82 ± 4.62) (*p* < 0.01). Both the vitamin B group (25.69 ± 2.63) and the vitamin B + ALA group (21.68 ± 2.69) had significantly reduced urea levels compared to the IRI group (*p* < 0.05). The IRI group had elevated creatinine levels (0.59 ± 0.07) compared to the sham group (0.45 ± 0.07) (*p* < 0.01). The vitamin B + ALA group (0.54 ± 0.05) showed a significant reduction in creatinine levels compared to the IRI group (*p* < 0.05). The LDH levels were significantly higher in the IRI group (2236.57 ± 890.05) compared to the sham group (406.28 ± 189.22) (*p* < 0.01). Both the vitamin B group (1157.38 ± 239.61) and the vitamin B + ALA group (978.25 ± 138.22) exhibited significantly lower LDH levels compared to the IRI group (*p* < 0.05), with the vitamin B + ALA group also showing a significant difference when compared to the sham group (*p* < 0.01).

The oxidative stress parameters measured in the study showed significant differences among the groups. The results are summarized in Table 2. The TAS levels were similar in the sham (0.71 ± 0.17) and IRI (0.71 ± 0.10) groups. However, the vitamin B (0.86 ± 0.16) and vitamin B + ALA (1.06 ± 0.41) groups demonstrated significantly higher TAS levels compared to both the sham and IRI groups (*p* < 0.01). The TOS levels were significantly elevated in the IRI group (104.78 ± 32.89) compared to the sham group (74.42 ± 63.24) (*p* < 0.05). Both the vitamin B (49.88 ± 12.81) and vitamin B + ALA (31.21 ± 22.46) groups showed significantly lower TOS levels compared to the IRI group (*p* < 0.01). The OSI, which is calculated from TAS and TOS, was significantly higher in the IRI group (145.72 ± 41.75) compared to the sham group (101.00 ± 88.74) (*p* < 0.05). The vitamin B (59.60 ± 21.33) and vitamin B + ALA (34.24 ± 34.34) groups exhibited significantly lower OSI values compared to the IRI group (*p* < 0.01), and both groups also showed significant improvements compared to the sham group (*p* < 0.05).

**Figure 1:**
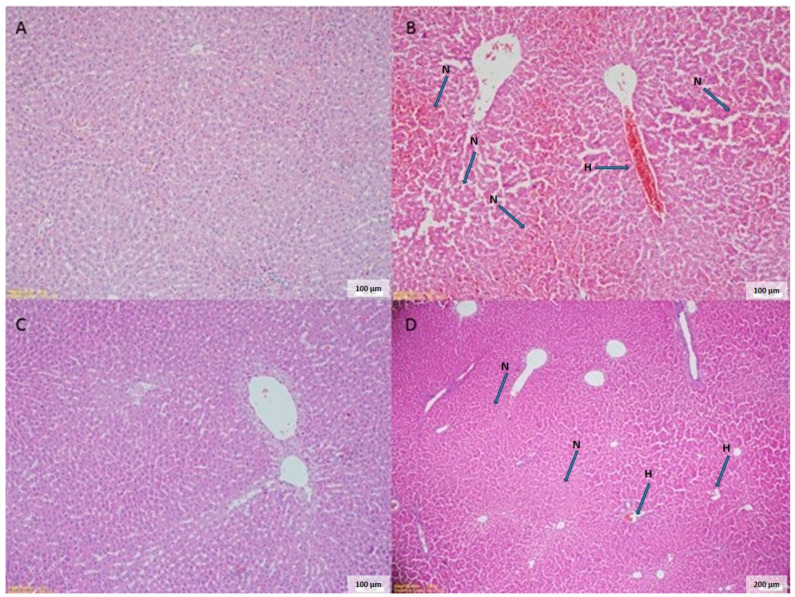
Histopathological findings (100×) of the study groups. Part (**A**) represents the sham group (control); Part (**B**) represents the severe necrotic (N), degenerative differentiations and severe hemorrhagic area (H) in the IRI group; Part (**C**) represents the vitamin B complex treated group; Part (**D**) represents the moderate necrotic (N) and degenerative differentiation and moderate hemorrhagic area (H) in the vitamin B + ALA treated IRI group. Kruskal–Wallis test (post hoc Mann–Whitney U test with Bonferroni correction).

The histopathological comparisons of rat livers among the different groups showed significant differences, as summarized in Table 3. The IRI group exhibited significantly higher levels of cellular swelling (2.75 ± 0.2) compared to the sham group (1.47 ± 0.2) (*p* < 0.01). Both the vitamin B (1.10 ± 0.0) and vitamin B + ALA (1.60 ± 0.1) groups showed significant reductions in cellular swelling compared to the IRI group (*p* < 0.01). The level of congestion was significantly increased in the IRI group (3.65 ± 0.2) compared to the sham group (1.90 ± 0.3) (*p* < 0.01). The vitamin B (1.71 ± 0.1) and vitamin B + ALA (3.31 ± 0.1) groups exhibited significantly lower congestion levels compared to the IRI group (*p* < 0.05 and *p* < 0.01). The IRI group had significantly higher infiltration of polymorph nuclear leukocytes (PNL) (1.77 ± 0.3) compared to the sham group (1.18 ± 0.1) (*p* < 0.01). Both the vitamin B (1.14 ± 0.1) and vitamin B + ALA (1.10 ± 0.3) groups showed significant reductions in leukocyte infiltration compared to the IRI group (*p* < 0.01). Apoptosis levels were significantly higher in the IRI group (0.57 ± 0.2) compared to the sham group (0.28 ± 0.1) (*p* < 0.01). The vitamin B (0.14 ± 0.1) and vitamin B + ALA (0.34 ± 0.2) groups exhibited lower levels of apoptosis compared to the IRI group (*p* < 0.01 and *p* < 0.05). Histopathological findings (100x) of study groups are shown in Figure 1.

## 4. Discussion

In this study, we evaluated the impact of a vitamin B complex and a vitamin B complex combined with ALA on the histopathological outcomes and oxidative status markers in liver IRI in rats. Our findings indicate that both the vitamin B and vitamin B + ALA groups showed significantly better results across all biochemical parameters, oxidative status markers, and histopathological assessments compared to the IRI group. To the best of our knowledge, this is the first study to report the effects of preconditioning with either vitamin B complex or vitamin B complex + ALA on liver IRI in the literature.

Ischemia–reperfusion injury in any organ has been associated with the increased production of ROS. ROS accumulation directly results in oxidation of several molecules such as lipids, proteins, and DNA while it indirectly activates signaling pathways, causing cellular dysfunction and cell apoptosis [3]. Thus, suppressing ROS production is one of the main strategies in protection against IRI.

Alpha-lipoic acid (ALA) is a strong antioxidant with many direct and indirect effects including direct radical scavenging, recycling, and metal chelation, regeneration of endogenous antioxidants, improving endothelial function and accelerating glutathione synthesis [35]. ALA has been studied in the prevention of IRI with successful results in different tissues including brain, heart, testis and kidney [36,37,38,39]. In the literature there are also a few studies reporting the effects of ALA in IRI of the liver [24]. In an experimental study, Ren et al. reported that preconditioning with ALA significantly reduced LDH and purine nucleoside phosphorylase levels and significantly reduced post-ischemic activation of NF-kappa B during IRI of the rat liver. The authors concluded that ALA administration attenuates IRI [24]. Dunschede et al. reported for the first time that ATP content was significantly enhanced after 30 min of ischemia followed by 30 min of reperfusion of the liver in patients under preconditioning with ALA [40]. Dulundu et al. administered ALA [100 mg/kg) 15 min prior to ischemia and immediately before reperfusion to rats with IRI of the liver to determine its role in preventing oxidative damage and reported significantly increased hepatic glutathione levels and decreased malondialdehyde levels and myeloperoxidase activity in the ALA group [23]. Moreover, in that study, similar to ours, serum ALT, AST, and LDH activities were determined to be statistically significantly decreased in the ALA treated group compared with the IRI group. Supporting these studies, we also noted a significantly improved oxidative status and decreased cellular swelling and decreased number of PNL in the ALA administered group together with the vitamin B complex. In our study, although the oxidative status was better in the vitamin B +ALA compared with the vitamin B alone group; the differences were not statistically significant. This may be associated with the low dose of ALA used in our study and higher doses may be associated with better results, which would be the topic of another study.

In another experimental study, pretreatment with ALA was compared with different ischemia preconditioning strategies to protect the liver from IRI and decreased cellular damage. Decreased caspase 9-activity in the liver tissue and decreased serum lipid peroxidation levels were reported in the ALA group compared with the ischemia preconditioning and sham groups [24]. In that study, the authors also reported increased levels of anti-apoptotic proteins in the ALA group. Similarly, we have also determined decreased apoptotic activity in the vitamin B and vitamin B +ALA groups than the IRI group in histopathological evaluation but the differences were not statistically significant.

On the other hand, different vitamin B forms also are well known with their anti-oxidant effects and some of them were also studied before in IRI of different tissues [22,41]. However, the data about the effects of different vitamin B forms in hepatic IRI are limited. Recently, Sanches et al. investigated the effects of riboflavin on liver IRI and reported significantly reduced serum and histological parameters of hepatocellular damage, neutrophil infiltration and oxidative stress by riboflavin administration [41]. In another study, Chen et al. reported that niacinamide administration significantly attenuated liver injury with a reduction of methyl guanidine, tumor necrosis factor [TNF-alpha) and nitric oxide release [42]. Similar to our study, they also reported decreased serum ALT, AST and LDH levels in the niacinamide group compared to the IRI group. To the best of our knowledge, this is the first study evaluating the effects of vitamin B complex on IRI of rat liver and we have found significantly improved histopathological results and oxidative status parameters in the vitamin B complex administered group compared with the IRI group.

In ischemia–reperfusion injury of the liver, distant organ damage such as in kidney, heart, and lung is not a surprise due to the release of pro-inflammatory cytokines and oxygen-derived radicals into the circulation from damaged liver tissue [33,43]. However, to the best of our knowledge, the effects of a vitamin B complex or ALA on kidney functions during IRI of the liver have not been studied before. In this study, we have determined that creatinine levels were not statistically significantly different between all groups but urea levels were statistically significantly better in the vitamin B + ALA group than the IRI group, suggesting that ALA administration together with a vitamin B complex may improve kidney functions during IRI of the liver [44].

There are some limitations of the current study. First, this is a single center study. Second, another limitation of our study is lacking an in-depth mechanism for the protective effects of vitamin B and ALA; therefore, further parameters are required to support the hypothesis of the study. The strength of our study is that this is the first study to report the effects of preconditioning with either vitamin B complex or vitamin B complex + ALA on liver IRI in the literature.

## 5. Conclusions

In conclusion, our study demonstrated that the groups treated with vitamin B complex alone and in combination with ALA exhibited significantly improved histopathological outcomes and oxidative stress markers compared to the IRI group. Although the differences were not statistically significant, the vitamin B + ALA group showed more favorable biochemical results and oxidative status than the vitamin B group alone. The low dose of ALA used in this study may have contributed to these findings. Future research with higher doses of ALA is necessary to fully elucidate its potential impact on hepatic ischemia–reperfusion injury. Our results suggest that a vitamin B complex, either alone or combined with ALA, effectively mitigates post-ischemic liver injury by enhancing antioxidative capacity.

## Figures and Tables

**Table 1:** The Comparison of Biochemical Parameters among Groups.

	Sham (n = 10) Mean ± SD	IRI (n = 10) Mean ± SD	Vit-B (n = 10) Mean ± SD	Vit-B + ALA (n = 10) Mean ± SD
ALT (IU/dL)	35.42 ± 5.80	1549.85 ± 359.76 *	1013.12 ± 785.39 *, #	934.75 ± 158.80 *, ##
AST (IU/dL)	121.28 ± 44.35	1573.0 ± 1437.17 *	1193.37 ± 493.40 **, #	1002.62 ± 133.84 **, #
Urea (mg/dL)	18.82 ± 4.62	28.75 ± 2.98 *	25.69 ± 2.63 #	21.68 ± 2.69 ##
Creatinine (mg/dL)	0.45 ± 0.07	0.59 ± 0.07	0.56 ± 0.08	0.54 ± 0.05
LDH (IU/L)	406.28 ± 189.22	2236.57 ± 890.05 **	1157.38 ± 239.61 *, #	978.25 ± 138.22 **, #

ALT: Alanine amino transferase, AST: Aspartate amino transferase, LDH: Lactate dehydrogenase, IRI: Ischemia–reperfusion injury; Vit-B: Vitamin B complex; ALA: Alpha lipoic acid. *: compared with sham group; #: compared with IRI group. *: *p* < 0.05; **: *p* < 0.01. #: *p* < 0.05; ##: *p* < 0.01.

**Table 2:** The comparison of oxidative stress parameters among groups.

	Sham (n = 10) Mean ± SD	IRI (n = 10) Mean ± SD	Vit-B (n = 10) Mean ± SD	Vit-B + ALA (n = 10) Mean ± SD
TAS	0.71 ± 0.17	0.71 ± 0.10	0.86 ± 0.16 *, ##	1.06 ± 0.41 *, ##
TOS	74.42 ± 63.24	104.78 ± 32.89 *	49.88 ± 12.81 *, ##	31.21 ± 22.46 *, ##
OSI	101.00 ± 88.74	145.72 ± 41.75 *	59.60 ± 21.33 *, ##	34.24 ± 34.34 *, ##

IRI: Ischemia–reperfusion injury; Vit-B: Vitamin B complex; ALA: Alpha lipoic acid. *: compared with sham group; #: compared with IRI group. *: *p* < 0.05; ##: *p* < 0.01.

**Table 3:** The histopathological comparisons of rat livers among groups.

	Sham (n = 10) Mean ± SD	IRI (n = 10) Mean ± SD	Vit-B (n = 10) Mean ± SD	Vit-B + ALA (n = 10) Mean ± SD
**Cellular swelling**	1.47 ± 0.2	2.75 ± 0.2 *	1.10 ± 0.0 ##	1.60 ± 0.1 ##
**Congestion**	1.90 ± 0.3	3.65 ± 0.2 *	1.71 ± 0.1 ##	3.31 ± 0.1 #, ^$^
**Polymorphic nuclear leukocytes**	1.18 ± 0.1	1.77 ± 0.3 *	1.14 ± 0.1 ##	1.10 ± 0.3 ##
**Apoptosis**	0.28 ± 0.1	0.57 ± 0.2	0.14 ± 0.1 ##	0.34 ± 0.2 #

IRI: Ischemia–reperfusion injury; Vit-B: Vitamin B complex; ALA: Alpha lipoic acid. *: compared with sham group; #: compared with IRI group. ^$^: compared with Vit-B group. *: *p* < 0.05; #: *p* < 0.05; ##: *p* < 0.01. ^$^: *p* < 0.01. Kruskal–Wallis test (post hoc Mann–Whitney U test with Bonferroni correction).

## Data Availability

The data are available upon request to the corresponding author.
